# Antimicrobials use and infection hospital contacts as proxies of infection exposure at ages 0–2 years and risk of infectious mononucleosis

**DOI:** 10.1038/s41598-023-48509-3

**Published:** 2023-12-01

**Authors:** Klaus Rostgaard, Signe Holst Søegaard, Lone Graff Stensballe, Henrik Hjalgrim

**Affiliations:** 1 Danish Cancer Society Research Center, Danish Cancer Society, Copenhagen, Denmark; 2 Department of Epidemiology Research, Statens Serum Institut, Copenhagen, Denmark; 3 Department of Pediatrics and Adolescent Medicine, Rigshospitalet, Copenhagen University Hospital, Copenhagen, Denmark; 4 Department of Hematology, Copenhagen University Hospital, Copenhagen, Denmark; 5 Department of Clinical Medicine, Copenhagen University, Copenhagen, Denmark

**Keywords:** Diseases, Medical research, Risk factors

## Abstract

Infectious mononucleosis (IM) often results from late primary infection with Epstein–Barr virus (EBV). Exposure to EBV at ages 0–2 years from, e.g., siblings therefore protects against IM. Using Danish registers, we therefore followed children born in 1997 through 2015 from age 3 years for a hospital contact with an IM diagnosis as outcome with the number of antimicrobial prescriptions filled before age 3 years as a proxy of infection pressure and the main exposure in stratified Cox regressions. The main analyses used sibships as strata primarily to adjust for health-seeking behaviour with further possible adjustments for age, sex, calendar period and sibship constellation. In these analyses we followed 7087 children, exposed on average to 3.76 antimicrobials prescriptions. We observed a crude hazard ratio for IM per unit increase in cumulative antimicrobial use of 1.00 (95% confidence interval 0.99, 1.02), with similar results in adjusted analyses. The hypothesis that children with the largest use of antimicrobials at ages 0–2 years would subsequently have the lowest risk of IM within a sibship was not corroborated by the data. Furthermore, sibship-matched analyses provided no support for some common early-life immune system characteristics being predictive of IM.

## Introduction

Most individuals become infected with Epstein-Barr Virus (EBV), which then establishes a persistent mostly latent infection in the host^1^. The primary EBV infection usually occurs either in early childhood (ages 0–2 years) or during teenage years, with roughly a third of all individuals primary infected in each period, according to recent modeling in a contemporary Danish population^1^. Similar corresponding age- and sex-specific prevalences of EBV-infected individuals are found throughout the Western world, but with noteworthy variation by socio-demography, ethnicity and secular changes, such that the prevalence of EBV is increased in poor sociodemographic settings, in people of Asian or African descent, and in old times^2–9^. When the primary EBV infection occurs in teenage years or later it often presents as infectious mononucleosis (IM), while this presentation is rare at younger ages^1^.

Transmission of EBV is usually through saliva, which in teenagers and older individuals typically would occur through deep kissing. Meanwhile, it is not so clear why and how individuals are infected with EBV earlier in life^1,10,11^. Also, we do not understand why some individuals present with IM upon primary EBV infection, while others do not^1,10^. The public health consequences of IM by itself, and other sequelae of EBV infection including multiple sclerosis, other autoimmune diseases, and several cancer forms are sufficiently common and consequential to have spurred research into vaccination targeting EBV-associated biological pathways^12,13^. Most likely, EBV vaccination targeting multiple sclerosis and other long-term consequences of primary EBV infection, rendering randomised clinical trials infeasible, could only be developed and applied based on a consensus in the broad scientific community, that all relevant aetiological details were known, creating confidence that the vaccine would work as intended, and at least not harm in unforeseen ways^14^. Answering the above aetiological questions about IM would almost surely aid EBV vaccine research and design^15,16^, as necessary basic EBV research. Immediate public health effects of this research are unlikely, but the prospects of an EBV vaccine are profound, promising essentially the eradication of IM and multiple sclerosis^11^.

The association between IM/late primary EBV infection and several proxies of infection pressure in childhood (sibship constellation, birth order, sibship size, childcare,…) has been examined, but to the best of our knowledge, no-one has so far used prescribed antimicrobials as such a proxy^11,17^. Obviously, the prescribed antimicrobials would only represent (treatment of) the top of the iceberg of infections. Example, a Swedish community study found that for respiratory tract infections in 18-month-old children, only 6% were treated with antibiotics and only 13% led to a consultation with a general practitioner^18^. Furthermore, primary EBV infection in this age span is usually asymptomatic, so only in very rare cases would it be the EBV infection that provoked treatment with antimicrobials. But antimicrobials use may be a useful marker of behaviours and environments conducive to EBV transmission. While the aforementioned characteristics are clearly proxies of primarily exogenous exposures, we would expect antimicrobial use to a much larger extent to reflect host characteristics, primarily the functioning and maturation of the immune system, after having accounted for sociodemographic co-determinants of antimicrobial use^6,19–22^.

Accordingly, we would expect a priori that individuals diagnosed with IM in adolescence would more often also have escaped other infections in early life and that this would be reflected in a lower use of antimicrobials in these individuals than among comparators in early life. However, in analyses suitably adjusted for well-established proxies of environments conducive to EBV transmission, primarily sibship constellation^11, 23^, we would expect to end up characterizing mainly a proxy for host characteristics.

## Methods

### Cohort

As exposures, we assessed hospital contacts with infection and antimicrobial prescriptions in the first 3 years of life for children born in Denmark during the period 1997 to 2015 with known parents. If resident in Denmark throughout these first 3 years, these children were then followed for IM from age 3 years during the period 2000 to 2018 while resident in Denmark. We first performed analyses of this full cohort, secondly, we performed analyses nested in this cohort based exclusively on within-sibship comparisons to control for confounding by sociodemographic factors, sibships being defined by the unique combination of mother and father. Information on family relations, residence, place of birth, sex, birth dates, and civil statuses was obtained from the Danish Civil Registration System^24^, exposures and outcomes from Danish nationwide health registers^25^.

### Exposures

For all participants, we obtained information on antimicrobial prescriptions (ATC codes J01A, J01C-J01G, J01M, J01X, J02A, J04A, J05A, P01A, P02C) from the Danish Prescription Register, covering the period since 1996^26,27^. The prescriptions included antibacterials, antimycobacterials, antifungals, antivirals, antiprotozoals, and antihelmintics^28^. The vast majority of these products were intended for treating respiratory infections (ATC codes: J01CA04, J01CE02, J01CR02, J01FA)^29^. We likewise obtained information on hospital contacts involving main, secondary or underlying diagnoses for infectious diseases (ICD10 chapters A and B) from the Danish National Patient Register^30^. As exposures we tallied the number of such antimicrobial prescriptions and number of such hospital contacts during age 0 years, age 1 year, age 2 years and ages 0–2 years cumulated.

### Outcome

We identified IM cases as persons having a main, secondary, or underlying diagnosis code of B27 (ICD-10) in a hospital contact in the Danish National Patient Register^30^, taking the earliest admission date for such a contact to be the date of IM diagnosis.

### Statistical analysis

For each type of exposure (prescriptions or hospital contacts) we examined cumulative exposure (0–2 years) or age-specific exposure (0, 1, 2 years), on IM risk either overall (age 3+ years) or age-specific (3–12 years, 13+ years).

Sociodemographic factors adjusted for were mother’s age at birth of the index child as a log-linear trend, and for each parent whether they were born in a non-Western country, defined as being born outside Europe (excluding Turkey and including USA and Canada).

To model the effect of sibship constellation, we used the number of siblings of a certain age (0, 1, 2, 3 years) and number of siblings with a certain age differential to the index child in eight categories, and an interaction between the age of each sibling and the age of the index person as time-varying predictors, with siboffset = Σ_k_ log(HR_k_) × pred_k_ using the predictors and hazard ratios from Rostgaard et al*.*^11^.

In the first set of analyses, we used the full cohort and defined strata for the stratified Cox regression by combinations of sex and year of birth, with age as the underlying time scale. The three variants of analyses were crude, then adjusted for sociodemographic factors, and finally further adjusted for sibship constellation by adding the above siboffset as an offset.

In the second set of analyses we performed stratified Cox regressions, with calendar time as underlying time scale, and combinations of mothers and fathers defining the sibship strata. For each exposure combination we examined three models; a crude model, a model adjusted for age and sex through an offset, and finally a model further adjusted for sibship constellation by including the above siboffset in the offset. These offsets were time-dependent. The adjustments were performed via offsets to avoid technical problems and instabilities^11^. We calculated sex- and age-specific empirical IM incidence rates (events/pyrs) in our data, with age in 1-year categories and entering the log of the relevant rates as offsets.

All analyses were performed using the SAS statistical software package (version 9.4 SAS Institute, Cary, NC, USA). Ninety-five percent confidence intervals (CIs) were based on Wald tests.

### Ethics

This study was performed in accordance with the Declaration of Helsinki and was approved by SSI QA and Compliance (j.nr. 20/13012), the institutional review board of Statens Serum Institut (SSI), Copenhagen, Denmark. This permit also stipulates that (1) no informed consent was needed (basis for legal handling of data being General Data Protection Regulation article 6, 1e), (2) no special ethics permits were needed, and (3) the data supporting findings of the study are available upon reasonable request from KR (klar@cancer.dk). Thus, the need/requirement for informed consent was waived by SSI QA and Compliance for this study, in accordance with national legislation. Further, to obtain the data in this way at a minimum requires fulfillment of §10,1 and §10,2 in the Danish Data protection law, and following SSI’s procedures for obtaining data.

## Results

In the first set of analyses, our study cohort consisted of 595,237 boys and 565,434 girls followed for 11,174,676 person-years (mean follow-up 9.6 years) yielding 4770 IM events, 4167 (87%) of which were based on IM as a main diagnosis. On average 0.17 hospital contacts for infections and 2.95 antimicrobial prescriptions were observed during the first three years of life for the followed persons. The distribution of considered exposures are presented in Table 1.

**Table 1:** Counts of antimicrobial prescriptions and hospital contacts with infection according to age at exposure.

Exposure	0	%	1	%	2	%	3 +	%
	n		n		n		n	
Antimicrobials age 0 years	695,062	60	249,904	22	114,512	10	101,193	9
Antimicrobials age 1 year	482,229	42	281,018	24	170,135	15	227,289	20
Antimicrobials age 2 years	658,314	57	265,408	23	128,307	11	108,642	9
Antimicrobials age 0–2 years	252,377	22	222,290	19	180,230	16	505,774	44
Hospital contacts age 0 years	1,090,521	94	57,213	5	9919	1	3018	0
Hospital contacts age 1 year	1,101,831	95	48,925	4	7701	1	2214	0
Hospital contacts age 2 years	1,132,437	98	24,185	2	3215	0	834	0
Hospital contacts age 0–2 years	1,019,631	88	106,963	9	23,656	2	10,421	1

The observed effect sizes were all small but both use of antimicrobials and hospitalizations for infections were associated with increased risk of IM (Tables 2, 3).

**Table 2:** Hazard ratios (HRs) with 95% confidence interval (CI) for IM per additional hospital contact with infection estimated among 1,160,671 children (full cohort).

Age at follow-up (years)	Hospital contacts	HR^a^ (95% CI)	HR^b^ (95% CI)	HR^c^ (95% CI)
3–12	Age 0 years	1.31 (1.20, 1.44)	1.31 (1.19, 1.43)	1.31 (1.20, 1.44)
3–12	Age 1 year	1.28 (1.16, 1.41)	1.27 (1.16, 1.40)	1.27 (1.15, 1.40)
3–12	Age 2 years	1.16 (1.00, 1.34)	1.16 (1.00, 1.34)	1.16 (1.00, 1.34)
3–12	Age 0–2 years	1.26 (1.21, 1.32)	1.26 (1.20, 1.32)	1.26 (1.20, 1.32)
13+	Age 0 years	1.13 (1.00, 1.28)	1.17 (1.04, 1.32)	1.16 (1.03, 1.31)
13+	Age 1 year	1.27 (1.13, 1.43)	1.29 (1.14, 1.44)	1.28 (1.14, 1.44)
13+	Age 2 years	1.07 (0.87, 1.30)	1.09 (0.90, 1.33)	1.09 (0.90, 1.33)
13+	Age 0–2 years	1.17 (1.09, 1.26)	1.20 (1.12, 1.28)	1.20 (1.12, 1.28)
3+	Age 0 years	1.24 (1.15, 1.33)	1.25 (1.16, 1.34)	1.25 (1.16, 1.35)
3+	Age 1 year	1.28 (1.19, 1.38)	1.28 (1.19, 1.38)	1.28 (1.19, 1.38)
3+	Age 2 years	1.12 (0.99, 1.27)	1.14 (1.01, 1.28)	1.13 (1.00, 1.28)
3+	Age 0–2 years	1.23 (1.18, 1.28)	1.24 (1.19, 1.29)	1.24 (1.19, 1.29)

All analyses were stratified by sex and year of birth with age of the child as underlying time-scale.

^a^Crude, ^b^adjusted for mother’s age at birth and parental country of birth, ^c^further adjusted for sibship constellation (number of siblings, age differential to the index child, and an interaction between sibling’s age and age of index child, see text for modelling description).

**Table 3:** Hazard ratios (HRs) with 95% confidence interval (CI) for IM per additional prescribed antimicrobial estimated among 1,160,671 children (full cohort).

Age at follow-up (years)	Antimicrobials	HR^a^ (95% CI)	HR^b^ (95% CI)	HR^c^ (95% CI)
3–12	Age 0 years	1.07 (1.04, 1.09)	1.06 (1.04, 1.09)	1.07 (1.04, 1.09)
3–12	Age 1 year	1.04 (1.02, 1.07)	1.04 (1.02, 1.07)	1.04 (1.02, 1.06)
3–12	Age 2 years	1.03 (1.01, 1.06)	1.03 (1.01, 1.06)	1.03 (1.01, 1.06)
3–12	Age 0–2 years	1.04 (1.04, 1.05)	1.04 (1.04, 1.05)	1.04 (1.04, 1.05)
13+	Age 0 year	1.04 (1.01, 1.06)	1.04 (1.01, 1.07)	1.04 (1.01, 1.06)
13+	Age 1 year	1.06 (1.04, 1.08)	1.06 (1.04, 1.08)	1.06 (1.03, 1.08)
13+	Age 2 years	1.01 (0.99, 1.03)	1.01 (0.99, 1.03)	1.01 (0.99, 1.03)
13+	Age 0–2 years	1.03 (1.03, 1.04)	1.04 (1.03, 1.04)	1.03 (1.03, 1.04)
3+	Age 0 years	1.05 (1.03, 1.07)	1.05 (1.03, 1.07)	1.05 (1.03, 1.07)
3+	Age 1 year	1.05 (1.04, 1.07)	1.05 (1.03, 1.07)	1.05 (1.03, 1.06)
3+	Age 2 years	1.02 (1.00, 1.03)	1.02 (1.01, 1.04)	1.02 (1.01, 1.04)
3+	Age 0–2 years	1.04 (1.03, 1.04)	1.04 (1.03, 1.04)	1.04 (1.03, 1.04)

All analyses were stratified by sex and year of birth with age of the child as underlying time-scale.

^a^Crude, ^b^adjusted for mother’s age at birth and parental country of birth, ^c^further adjusted for sibship constellation (number of siblings, age differential to the index child, and an interaction between sibling’s age and age of index child, see text for modelling description).

In secondary analyses based exclusively on within-sibship comparisons the study cohort consisted of 3569 boys and 3518 girls from 3000 sibships followed for 74,313 person-years (mean follow-up 10.5 years) yielding 3026 IM events, 2613 (86%) of which were based on IM as a main diagnosis. On average 0.18 infectious hospital contacts and 3.76 antimicrobial prescriptions were observed during the first three years of life for the followed persons.

The observed effect sizes in within-sibship analyses were all very small and mostly guaranteed to be so by relatively narrow confidence intervals. Further, there was no suggestion of real variation in effect sizes by age at follow-up, age of exposure or degrees of confounder adjustment (Tables 4, 5). Considering the small variation in exposure between siblings, these small effect sizes did not predict meaningful variation in IM risk between siblings by antimicrobials use or infection hospital contacts at ages 0–2 years.

**Table 4:** Hazard ratios (HRs) with 95% confidence interval (CI) for IM per additional hospital contact with infection estimated among 7087 children from 3000 sibships.

Age at follow-up (years)	Hospital contacts	HR^a^ (95% CI)	HR^b^ (95% CI)	HR^c^ (95% CI)
3–12	Age 0 years	1.14 (0.90, 1.45)	1.09 (0.86, 1.39)	1.08 (0.85, 1.37)
3–12	Age 1 year	0.94 (0.74, 1.19)	0.94 (0.74, 1.20)	0.95 (0.75, 1.20)
3–12	Age 2 years	0.99 (0.65, 1.50)	1.02 (0.67, 1.56)	1.04 (0.68, 1.59)
3–12	Age 0–2 years	1.03 (0.89, 1.19)	1.01 (0.87, 1.18)	1.01 (0.87, 1.18)
13+	Age 0 years	1.00 (0.77, 1.30)	1.02 (0.77, 1.36)	1.01 (0.76, 1.34)
13+	Age 1 year	1.19 (0.83, 1.70)	1.19 (0.81, 1.73)	1.21 (0.83, 1.75)
13+	Age 2 years	0.92 (0.54, 1.56)	0.86 (0.49, 1.52)	0.84 (0.48, 1.48)
13+	Age 0–2 years	1.04 (0.86, 1.26)	1.04 (0.85, 1.28)	1.04 (0.85, 1.27)
3+	Age 0 years	1.04 (0.89, 1.21)	1.07 (0.91, 1.27)	1.06 (0.90, 1.25)
3+	Age 1 year	1.03 (0.87, 1.23)	1.04 (0.86, 1.26)	1.05 (0.87, 1.27)
3+	Age 2 years	0.89 (0.67, 1.20)	0.92 (0.67, 1.25)	0.92 (0.68, 1.26)
3+	Age 0–2 years	1.01 (0.91, 1.12)	1.04 (0.93, 1.16)	1.03 (0.93, 1.15)

All analyses were stratified by sibship with calendar time as underlying time-scale.

^a^Crude, ^b^adjusted for age and sex, ^c^further adjusted for sibship constellation (number of siblings, age differential to the index child, and an interaction between sibling’s age and age of index child, see text for modelling description).

**Table 5:** Hazard ratios (HRs) with 95% confidence interval (CI) for IM per additional prescribed antimicrobial estimated among 7087 children from 3000 sibships.

Age at follow-up (years)	Antimicrobials	HR^a^ (95% CI)	HR^b^ (95% CI)	HR^c^ (95% CI)
3–12	Age 0 years	0.97 (0.91, 1.04)	1.01 (0.94, 1.08)	1.00 (0.93, 1.07)
3–12	Age 1 year	1.01 (0.96, 1.06)	1.00 (0.95, 1.06)	1.00 (0.95, 1.06)
3–12	Age 2 years	1.03 (0.97, 1.10)	1.03 (0.96, 1.10)	1.03 (0.96, 1.10)
3–12	Age 0–2 years	1.00 (0.98, 1.03)	1.01 (0.98, 1.04)	1.01 (0.98, 1.04)
13+	Age 0 years	1.01 (0.94, 1.08)	0.99 (0.92, 1.06)	0.98 (0.91, 1.05)
13+	Age 1 year	0.99 (0.94, 1.04)	0.99 (0.94, 1.05)	0.99 (0.94, 1.05)
13+	Age 2 years	1.00 (0.94, 1.06)	0.99 (0.93, 1.06)	0.99 (0.93, 1.05)
13+	Age 0–2 years	1.00 (0.97, 1.02)	0.99 (0.96, 1.02)	0.99 (0.96, 1.02)
3+	Age 0 years	0.99 (0.95, 1.04)	1.01 (0.96, 1.05)	1.00 (0.96, 1.04)
3+	Age 1 year	1.01 (0.98, 1.04)	1.00 (0.97, 1.03)	1.00 (0.97, 1.04)
3+	Age 2 years	1.01 (0.97, 1.05)	1.01 (0.96, 1.05)	1.00 (0.96, 1.05)
3+	Age 0–2 years	1.00 (0.99, 1.02)	1.00 (0.98, 1.02)	1.00 (0.98, 1.02)

All analyses were stratified by sibship with calendar time as underlying time-scale.

^a^Crude, ^b^adjusted for age and sex, ^c^further adjusted for sibship constellation (number of siblings, age differential to the index child, and an interaction between sibling’s age and age of index child, see text for modelling description).

The small variation in results by varying degrees of confounder adjustment prompted us to assess the correlations between exposures and some potential confounders as well as within exposures and potential confounders (Supplementary Tables S1–S3). The correlations between exposures and various proxies of exposure were vanishingly small and therefore at a technical level explaining the minimal effect of confounder adjustment. On the other hand, the correlations between exposures were consistently positive and large enough to convince us that they were all measurements of the same phenomena.

The unexpected sign of the effect sizes in Tables 2 and 3 prompted us to redo the analyses restricted to the sub cohort of children with no siblings before age 5 years in an attempt to avoid confounding by sibship dynamics. However, the results (Supplementary Tables S4, S5) were remarkably similar to the results in Tables 2 and 3.

Finally, we assessed the potential of an exposure “misclassification” to have deflated our findings towards the null. Specifically, we assessed the consequences of restricting antimicrobials to antivirals (ATC code J05A) (Supplementary Table S6). We found the results similar to the results in Table 3, but inevitably, with wider confidence intervals.

## Discussion

### Principal findings

We used Danish nationwide registers to “test” the general assumption that diagnosis of IM (in adolescence) would be a marker of reduced exposure to infections early in life. Contrary to this we found no noteworthy association between IM risk and antimicrobial use or infection hospital contacts at ages 0–2 years when implicitly adjusting for sociodemographic factors implied by a common sibship. Thus, the expectation or hypothesis that the children with the largest use of antimicrobials at ages 0–2 years would subsequently have the lowest risk of IM within a sibship was not corroborated by the data; neither was the corollary that adjusting for sibship constellation should shrink this effect towards the null. Furthermore, the absence of an association between antimicrobial use and IM risk within sibships suggests that there are no common immune system characteristics in early life affecting risk of IM or late primary EBV infection. These inferences were not challenged by varying exposure definitions in sensitivity analyses. Neither were they challenged by previous study findings; to the best of our knowledge, this is the first study to address the association between these infection proxies and IM. The closest thing to our study is probably Jansen et al., who among other things assessed the association between having had respiratory and/or gastric tract infections in the first year of life and being infected by EBV at age 6 years^9,17^. In accordance with our findings they also found no association between these childhood infections and later primary EBV-infection^9^.

### Strengths of the study

We performed a purely register-based study in nationwide complete registers, thus by design minimizing biases regarding recall, participation, exposure, outcome and follow-up. Access to hospital is free in Denmark, avoiding some trivial biases in the ascertainment of outcome and exposure^30^. In the secondary analyses biases from sociodemographic factors (including parental ethnicity/genetics) and secular trends were further mitigated by stratification on sibships. Some analyses were adjusted for sibship constellation, which we believe mediates much of the effect of other sociodemographic factors^23^. The narrow confidence intervals around the effect estimates for the antimicrobial exposures secured the statistical interpretation that variation in recorded exposure to infections at ages 0–2 years did not meaningfully affect the risk of IM.

### Limitations of the data

We have previously found the association of sibship constellation, another proxy of childhood infections, with hospitalized IM and self-reported IM, respectively, to be very similar^23^. This may not be true in the present analyses, since both use of antimicrobials and hospital contacts may be indicative of health seeking behaviour and increased health surveillance^19–21^, making it more likely that our exposures may appear positively associated with the risk of hospitalized IM. This may be the main cause for the difference in results between the full cohort and the sibship-matched analyses. There exists no study on the validity of IM diagnoses in the National Patient Register, but based on previous experience the fraction of false-positives is likely to be substantial^30,31^. This would typically bias the observed associations towards the null absent other biases controlled in the sibship-matched analyses.

We used antimicrobial prescriptions as a proxy for infections in general, i.e., bacterial as well as the more common viral infections, and under a tacit assumption that the number of antimicrobial prescriptions would be proportional to but much smaller than the true number of all infections. Example, a Swedish community study found that for respiratory tract infections in 18-month-old children, only 6% were treated with antibiotics and only 13% led to a consultation with a general practitioner^18^. In the present context, the proportionality assumption was supported by the close seasonal correlation between viral respiratory infections and antibiotics use in Danish children^32,33^. Our general knowledge about the association between the number of childhood infections and proxies thereof is limited^34^, but most studies find a clear association between the number of infections in childhood and the number (or presence) of (older) siblings^34–39^. We also found childcare enrolment to be associated with a marked transient increase in the use of antimicrobials^29^. Use of antimicrobials in childhood has also been associated with e.g. acute lymphoblastic leukemia^40^, type 1 diabetes^41^, asthma^42^, and Hodgkin lymphoma^43^, the latter recognized as clearly EBV-associated^43^. In the present study, it was therefore surprising to us to find very modest and sometimes negative correlations between antimicrobial use and other proxies of exposure to infections in childhood such as the number of older (rho = 0.03) and younger siblings (rho =  − 0.04), raising doubt about the validity of the proportionality assumption. Kinlaw et al*.*^33^ studied the risk of having an antibiotic prescribed in the first year of living in Denmark in 2004–2012. They found a marked increase in this 1-year risk from 35 to 43% going from having no (older) siblings to having more (older) siblings, but really no variation between having one or more (older) siblings. This finding is alluded to with smaller statistical precision elsewhere^44–46^. This suggests that there are many things other than infections per se to determine whether antimicrobials are prescribed for children, and that we therefore should not expect more than a roughly monotone relationship between the number of infections and the number of antimicrobial prescriptions.

### Interpretation

Theoretically, the very small observed within-sibship effect sizes could be the net effect of two much larger opposing effects, e.g. that some immune characteristic firstly would lead to more EBV sero conversions at an early age, thus lowering IM risk by preventing EBV sero conversion at an older age, and secondly make it likelier to cause IM if the primary EBV infection occurred in teenage-years or later. However, such a fine-tuned balancing of effects seems rather improbable and is not expected a priori in any way. Likewise, a balancing of the effect of external infection pressure and characteristics of the immune system seems improbable.

So, a much more probable interpretation of our findings is that neither a generally increased exposure to all sorts of infectious agents, nor some common trajectory of immune system development in the first 3 years of life is predictive of any part of the sequence of events leading to IM.

Our null result is well-aligned with our previous finding that forwarding childcare attendance by as much as a year only diminished IM risk slightly, while at the same time childcare enrolment was associated with a marked transient increase in the use of antimicrobials, not compensated later in life^29,47^. Other conceivable markers of infections or immune stimulation in early childhood including breastfeeding and caesarean section have not been found to be associated with EBV sero status^4,9,17,48–50^. Our results reinforce the impression from genetic and other studies that with the possible exception of IL-10 genotypes, there are no common immune system characteristics affecting the risk of IM or late primary EBV infection^6,51^. As such it suggests that the main biological mechanisms causally affecting IM risk are due to random phenomena that we are not aware of yet, say, whether the EBV immune response to primary infection was mainly due to T-cells or NK-cells which depends on age^11,52–54^, on top of age-dependent behavioural and environmental changes affecting the risk of exposure to EBV^1^. And further that these age-dependent behavioural and environmental changes of relevance to EBV are not to any noteworthy degree correlated with similar changes affecting the risk of the mainly respiratory infections we are measuring through use of antimicrobials in the first years of life^29^. This could occur if the relevant behaviour and environment was shared by practically all children.

The lack of dependence of IM risk on infection pressure in the first 3 years of life also supports the idea that it should be safe to administer a prophylactic EBV vaccine at this age, rather than postpone it to just before puberty as e.g. the HPV vaccine^11,55^.

## Conclusions

The expectation or hypothesis that the children with the largest use of antimicrobials at ages 0–2 years would subsequently have the lowest risk of IM within a sibship was not corroborated by the data; neither was the corollary that adjusting for sibship constellation should shrink this effect towards the null. In sibship-matched analyses we found no association between use of antimicrobials early in life and later risk of IM reinforcing the impression from other studies that there are no common immune system characteristics in early life affecting risk of IM or late primary EBV infection.

### Supplementary Information

Supplementary Tables.

## Data Availability

The data supporting findings of the study are available upon reasonable request from KR (klar@cancer.dk). To obtain them at a minimum requires fulfilment of §10,1 and §10,2 in the Danish Data protection law, and following SSI’s procedures for obtaining data.
