# Supplementary material for: Antimicrobials use and infection hospital contacts as proxies of infection exposure at ages 0–2 years and risk of infectious mononucleosis
Source: Sci Rep. 2023 Dec 1;13:21251. doi: 10.1038/s41598-023-48509-3 (PMC10692188; doi:10.1038/s41598-023-48509-3)
Supplement: Supplementary file 1 — Supplementary Tables. [file 41598_2023_48509_MOESM1_ESM.pdf]

Supplementary material

for

Antimicrobials use and infection hospital contacts as proxies of infection exposure at ages 0-2 years and risk of infectious mononucleosis

Klaus Rostgaard[1,2*], Signe Holst Søegaard[1,2], Lone Graff Stensballe[3] & Henrik Hjalgrim[1,2,4,5]

[1]Danish Cancer Society Research Center, Danish Cancer Society, Copenhagen, Denmark

[2]Department of Epidemiology Research, Statens Serum Institut, Copenhagen, Denmark

[3]Department of Pediatrics and Adolescent Medicine, Rigshospitalet, Copenhagen University Hospital, Copenhagen, Denmark

[4]Department of Hematology, Copenhagen University Hospital, Copenhagen, Denmark

[5]Department of Clinical Medicine, Copenhagen University, Copenhagen, Denmark

* E-mail: klar@cancer.dk

Supplementary Table S1

Crosstabulation of the main exposures

The FREQ Procedure

Frequency
Percent
Row Pct
Col Pct

Table of antibiotcum2 by NPRinfcum2					
	NPRinfcum2				
antibiotcum2	0	1	2	3+	Total
0	234464<br>20.20<br>92.90<br>22.99	14804<br>1.28<br>5.87<br>13.84	2390<br>0.21<br>0.95<br>10.10	719<br>0.06<br>0.28<br>6.90	252377<br>21.74
1	201514<br>17.36<br>90.65<br>19.76	16629<br>1.43<br>7.48<br>15.55	3141<br>0.27<br>1.41<br>13.28	1006<br>0.09<br>0.45<br>9.65	222290<br>19.15
2	160157<br>13.80<br>88.86<br>15.71	15754<br>1.36<br>8.74<br>14.73	3168<br>0.27<br>1.76<br>13.39	1151<br>0.10<br>0.64<br>11.05	180230<br>15.53
3	119930<br>10.33<br>87.33<br>11.76	13364<br>1.15<br>9.73<br>12.49	2851<br>0.25<br>2.08<br>12.05	1187<br>0.10<br>0.86<br>11.39	137332<br>11.83
4	88173<br>7.60<br>85.89<br>8.65	10888<br>0.94<br>10.61<br>10.18	2511<br>0.22<br>2.45<br>10.61	1082<br>0.09<br>1.05<br>10.38	102654<br>8.84
5	63228<br>5.45<br>84.49<br>6.20	8678<br>0.75<br>11.60<br>8.11	1989<br>0.17<br>2.66<br>8.41	937<br>0.08<br>1.25<br>8.99	74832<br>6.45
6	44773<br>3.86<br>83.21<br>4.39	6622<br>0.57<br>12.31<br>6.19	1668<br>0.14<br>3.10<br>7.05	746<br>0.06<br>1.39<br>7.16	53809<br>4.64
7+	107392<br>9.25<br>78.30<br>10.53	20224<br>1.74<br>14.75<br>18.91	5938<br>0.51<br>4.33<br>25.10	3593<br>0.31<br>2.62<br>34.48	137147<br>11.82
Total	1019631<br>87.85	106963<br>9.22	23656<br>2.04	10421<br>0.90	1160671<br>100.00

Summary Statistics for antibiotcum2 by NPRinfcum2

Cochran-Mantel-Haenszel Statistics (Based on Table Scores)				
Statistic	Alternative Hypothesis	DF	Value	Prob
1	Nonzero Correlation	1	23241.4498	<.0001
2	Row Mean Scores Differ	7	24148.3507	<.0001
3	General Association	21	24836.5373	<.0001

Supplementary Table S2
Spearman correlation internally among exposures, measured at age 3 years.

Variable	antibiot0	antibiot1	antibiot2	antibiotcum2	NPRinf0	NPRinf1	NPRinf2	NPRinfcum2
antibiot0	1.00	0.29	0.19	0.61	0.09	0.04	0.03	0.09
antibiot1	0.29	1.00	0.30	0.80	0.06	0.09	0.05	0.11
antibiot2	0.19	0.30	1.00	0.64	0.05	0.06	0.07	0.09
antibiotcum2	0.61	0.80	0.64	1.00	0.09	0.09	0.07	0.13
NPRinf0	0.09	0.06	0.05	0.09	1.00	0.09	0.06	0.69
NPRinf1	0.04	0.09	0.06	0.09	0.09	1.00	0.08	0.63
NPRinf2	0.03	0.05	0.07	0.07	0.06	0.08	1.00	0.43
NPRinfcum2	0.09	0.11	0.09	0.13	0.69	0.63	0.43	1.00

Supplementary Table S3
Spearman correlation between exposures and confounders, measured at age 3 years.
Xld= number of older sibling, yng=number of younger siblings, nwfather is an indicator variable that the father is born in a non-European country, similarly for nwmother, see text for definitions. Siboffsetdirect is the component of the siboffset modeling direct contagion, siboffsetindirect is the component of the siboffset modeling the longterm protective effect of siblings, see text.

Variable	Antibiot cum2	NPRinf cum2	xld	yng	Sib lings	Siboffse tindirect	Siboffset direct	agemom	nwfather	nwmother
antibiotcum2	1.00	0.13	0.03	-0.04	-0.00	0.04	-0.05	-0.05	0.02	-0.00
NPRinfcum2	0.13	1.00	-0.01	-0.01	-0.02	0.02	-0.00	-0.03	0.05	0.04
xld	0.03	-0.01	1.00	-0.34	0.82	-0.27	-0.26	0.40	0.10	0.08
yng	-0.04	-0.01	-0.34	1.00	0.22	-0.75	0.94	-0.20	0.02	0.01
siblings	-0.00	-0.02	0.82	0.22	1.00	-0.72	0.27	0.31	0.11	0.09
siboffsetindirect	0.04	0.02	-0.27	-0.75	-0.72	1.00	-0.76	-0.01	-0.06	-0.04
siboffsetdirect	-0.05	-0.00	-0.26	0.94	0.27	-0.76	1.00	-0.17	0.02	0.01
agemom	-0.05	-0.03	0.40	-0.20	0.31	-0.01	-0.17	1.00	-0.06	-0.03
nwfather	0.02	0.05	0.10	0.02	0.11	-0.06	0.02	-0.06	1.00	0.66
nwmother	-0.00	0.04	0.08	0.01	0.09	-0.04	0.01	-0.03	0.66	1.00

Supplementary Table S4.
Hazard ratios (HRs) for IM per additional hospital contact for children with no siblings in the first 5 years of life.
CI0=crude, CI1=adjusted, CI2=further adjusted.

Age at follow-up	Hospital contacts	ci0	ci1	ci2
3-12 years	age 0 years	1.07 (0.74, 1.56)	1.05 (0.72, 1.52)	1.05 (0.72, 1.52)
3-12 years	age 1 year	1.41 (1.06, 1.87)	1.41 (1.06, 1.87)	1.41 (1.06, 1.87)
3-12 years	age 2 years	0.98 (0.53, 1.80)	0.98 (0.53, 1.81)	0.98 (0.53, 1.81)
3-12 years	age 0-2 years	1.20 (1.01, 1.43)	1.19 (1.00, 1.41)	1.19 (1.00, 1.42)
13+ years	age 0 years	1.19 (0.88, 1.60)	1.22 (0.90, 1.64)	1.21 (0.90, 1.63)
13+ years	age 1 year	1.32 (1.04, 1.68)	1.33 (1.05, 1.69)	1.33 (1.05, 1.69)
13+ years	age 2 years	1.26 (0.88, 1.79)	1.28 (0.90, 1.81)	1.28 (0.90, 1.81)
13+ years	age 0-2 years	1.26 (1.11, 1.44)	1.28 (1.13, 1.47)	1.28 (1.12, 1.46)
3+ years	age 0 years	1.14 (0.90, 1.43)	1.14 (0.90, 1.43)	1.14 (0.90, 1.43)
3+ years	age 1 year	1.35 (1.13, 1.63)	1.36 (1.13, 1.63)	1.36 (1.13, 1.63)
3+ years	age 2 years	1.16 (0.85, 1.58)	1.17 (0.86, 1.59)	1.17 (0.86, 1.59)
3+ years	age 0-2 years	1.24 (1.12, 1.37)	1.24 (1.12, 1.38)	1.24 (1.12, 1.38)

Supplementary Table S5.
Hazard ratios (HRs) for IM per additional prescribed antimicrobial for children with no siblings in the first 5 years of life.
CI0=crude, CI1=adjusted, CI2=further adjusted.

Age at follow-up	antimicrobials	ci0	ci1	ci2
3-12 years	age 0 years	1.09 (1.00, 1.19)	1.08 (0.99, 1.18)	1.08 (0.99, 1.18)
3-12 years	age 1 year	0.99 (0.93, 1.07)	0.99 (0.93, 1.07)	0.99 (0.93, 1.07)
3-12 years	age 2 years	1.10 (1.02, 1.18)	1.10 (1.02, 1.18)	1.10 (1.02, 1.18)
3-12 years	age 0-2 years	1.05 (1.02, 1.08)	1.05 (1.02, 1.08)	1.05 (1.02, 1.08)
13+ years	age 0 years	1.01 (0.94, 1.08)	1.01 (0.94, 1.08)	1.01 (0.94, 1.08)
13+ years	age 1 year	1.09 (1.04, 1.14)	1.09 (1.03, 1.14)	1.09 (1.03, 1.14)
13+ years	age 2 years	1.01 (0.95, 1.08)	1.01 (0.95, 1.08)	1.01 (0.95, 1.08)
13+ years	age 0-2 years	1.04 (1.02, 1.07)	1.04 (1.02, 1.06)	1.04 (1.02, 1.06)
3+ years	age 0 years	1.03 (0.98, 1.09)	1.03 (0.97, 1.09)	1.03 (0.97, 1.09)
3+ years	age 1 year	1.06 (1.01, 1.10)	1.05 (1.01, 1.10)	1.05 (1.01, 1.10)
3+ years	age 2 years	1.04 (0.99, 1.10)	1.04 (0.99, 1.10)	1.04 (0.99, 1.10)
3+ years	age 0-2 years	1.05 (1.03, 1.06)	1.05 (1.03, 1.06)	1.05 (1.03, 1.06)

Supplementary Table S6.
Hazard ratios (HRs) for IM per additional prescribed antiviral for children with no siblings in the first 5 years of life.
CI0=crude, CI1=adjusted, CI2=further adjusted.

Age at follow-up	antivirals	ci0	ci1	ci2
3-12 years	age 0 years	0.97 (0.50, 1.91)	0.98 (0.52, 1.83)	0.98 (0.52, 1.84)
3-12 years	age 1 year	1.21 (0.86, 1.70)	1.25 (0.90, 1.75)	1.25 (0.89, 1.74)
3-12 years	age 2 years	0.92 (0.61, 1.41)	0.95 (0.63, 1.43)	0.95 (0.63, 1.43)
3-12 years	age 0-2 years	1.05 (0.86, 1.29)	1.08 (0.90, 1.29)	1.07 (0.90, 1.28)
13+ years	age 0 years	0.93 (0.57, 1.52)	0.93 (0.57, 1.52)	0.93 (0.57, 1.52)
13+ years	age 1 year	1.15 (0.85, 1.56)	1.15 (0.85, 1.56)	1.15 (0.85, 1.57)
13+ years	age 2 years	1.14 (0.94, 1.39)	1.14 (0.94, 1.38)	1.14 (0.94, 1.38)
13+ years	age 0-2 years	1.09 (1.00, 1.19)	1.09 (1.00, 1.19)	1.09 (1.00, 1.19)
3+ years	age 0 years	0.94 (0.64, 1.38)	0.94 (0.65, 1.35)	0.93 (0.65, 1.35)
3+ years	age 1 year	1.18 (0.94, 1.47)	1.19 (0.95, 1.49)	1.19 (0.95, 1.49)
3+ years	age 2 years	1.08 (0.88, 1.33)	1.08 (0.89, 1.33)	1.08 (0.88, 1.33)
3+ years	age 0-2 years	1.08 (0.99, 1.18)	1.09 (1.00, 1.18)	1.09 (1.00, 1.18)
